# Comparative performance of the 16S rRNA gene in DNA barcoding of amphibians

**DOI:** 10.1186/1742-9994-2-5

**Published:** 2005-03-16

**Authors:** Miguel Vences, Meike Thomas, Arie van der Meijden, Ylenia Chiari, David R Vieites

**Affiliations:** 1 Institute for Biodiversity and Ecosystem Dynamics, Zoological Museum, University of Amsterdam, Mauritskade 61, 1092 AD Amsterdam, The Netherlands; 2 Institute for Genetics, Evolutionary Genetics, University of Cologne, Weyertal 121, 50931 Köln, Germany; 3 Department of Biology (Evolutionary Biology), University of Konstanz, 78457 Konstanz, Germany; 4 Department of Integrative Biology, Museum of Vertebrate Zoology, 3101 Valley Life Sciences Bldg., University of California, Berkeley, CA 94720-3160, USA

## Abstract

**Background:**

Identifying species of organisms by short sequences of DNA has been in the center of ongoing discussions under the terms DNA barcoding or DNA taxonomy. A C-terminal fragment of the mitochondrial gene for *cytochrome oxidase subunit I *(*COI*) has been proposed as universal marker for this purpose among animals.

**Results:**

Herein we present experimental evidence that the mitochondrial *16S rRNA *gene fulfills the requirements for a universal DNA barcoding marker in amphibians. In terms of universality of priming sites and identification of major vertebrate clades the studied *16S *fragment is superior to *COI*. Amplification success was 100% for *16S *in a subset of fresh and well-preserved samples of Madagascan frogs, while various combination of *COI *primers had lower success rates.*COI *priming sites showed high variability among amphibians both at the level of groups and closely related species, whereas *16S *priming sites were highly conserved among vertebrates. Interspecific pairwise *16S *divergences in a test group of Madagascan frogs were at a level suitable for assignment of larval stages to species (1–17%), with low degrees of pairwise haplotype divergence within populations (0–1%).

**Conclusion:**

We strongly advocate the use of *16S rRNA *as standard DNA barcoding marker for vertebrates to complement *COI*, especially if samples *a priori *could belong to various phylogenetically distant taxa and false negatives would constitute a major problem.

## Background

The use of short DNA sequences for the standardized identification of organisms has recently gained attention under the terms DNA barcoding or DNA taxonomy [1-3]. Among the promising applications of this method are the assignments of unknown life-history stages to adult organisms [4,5], the large-scale identification of organisms in ecological or genomic studies [1,6] and, most controversially, explorative studies to discover potentially undescribed "candidate" species [4,7,8]. Although it is not a fundamentally new technique [9], DNA barcoding is promising because technical progress has made its large-scale, automated application feasible [3,6] which may accelerate taxonomic progress [10].

Although not necessarily under the specific concepts of DNA barcoding and DNA taxonomy, the diagnosis and even definition of taxa by their DNA sequences are realities in many fields and organism groups, such as prokaryotes, fungi, and soil invertebrates [1,6]. To use this approach on a large and formalized scale, consensus of the scientific community is essential with respect to the most suitable genes that allow robust and repeatable amplification and sequencing, and that provide unequivocal resolution to identify a broad spectrum of organisms. While D. Tautz and co-workers [3] proposed the nuclear ribosomal RNA genes for this purpose, P. D. N. Hebert and colleagues have strongly argued in favor of a 5' fragment of the mitochondrial gene for cytochrome oxidase, subunit I (*COI *or *COXI*) [2,11]. This gene fragment has been shown to provide a sufficient resolution and robustness in some groups of organisms, such as arthropods and, more recently, birds [2,4,7,11].

A genetic marker suitable for DNA barcoding needs to meet a number of criteria [2]. First, in the study group, it needs to be sufficiently variable to discriminate among most species, but sufficiently conserved to be less variable within than between species. Second, priming sites need to be sufficiently conserved to permit a reliable amplification without the risk of false negatives when the goal is the analysis of pooled samples, e.g. when the total of invertebrates from a soil sample is to be studied without separating individuals, or of environmental DNA such as subfossil DNA remains from the soil [12,13]. Third, the gene should convey sufficient phylogenetic information to assign species to major taxa using simple phenetic approaches. Fourth, its amplification and sequencing should be as robust as possible, also under variable lab conditions and protocols. Fifth, sequence alignment should be possible also among distantly related taxa.

Here we explore the performance of a fragment of the 16S ribosomal RNA gene (*16S*) in DNA barcoding of amphibians. As a contribution to the discussion about suitable standard markers we provide experimental data on comparative amplification success of *16S *and *COI *in amphibians, empirical data on conservedness of priming sites, and an example from the *16S*-based identification of amphibian larval stages.

## Results

### Amplification experiments

We performed independent amplification experiments with one set of *16S *primers and three published sets of *COI *primers [2,7] focusing on representatives of different frog, salamander and caecilian genera. The experiments were concordant in yielding more reliable and universal amplifications for *16S *than *COI*. In a set of fresh and well-preserved samples from relatively closely related mantellid frogs from Madagascar (Table 1, Additional file 1), the *16S *amplification success was complete, whereas the three sets of *COI *primers yielded success rates of only 50–70%. Considering all three primer combinations, there were two species of frogs (10%) that did not amplify for *COI *at all (*Boophis septentrionalis *and *B. tephraeomystax*).

### Priming sites

**Figure 1:**
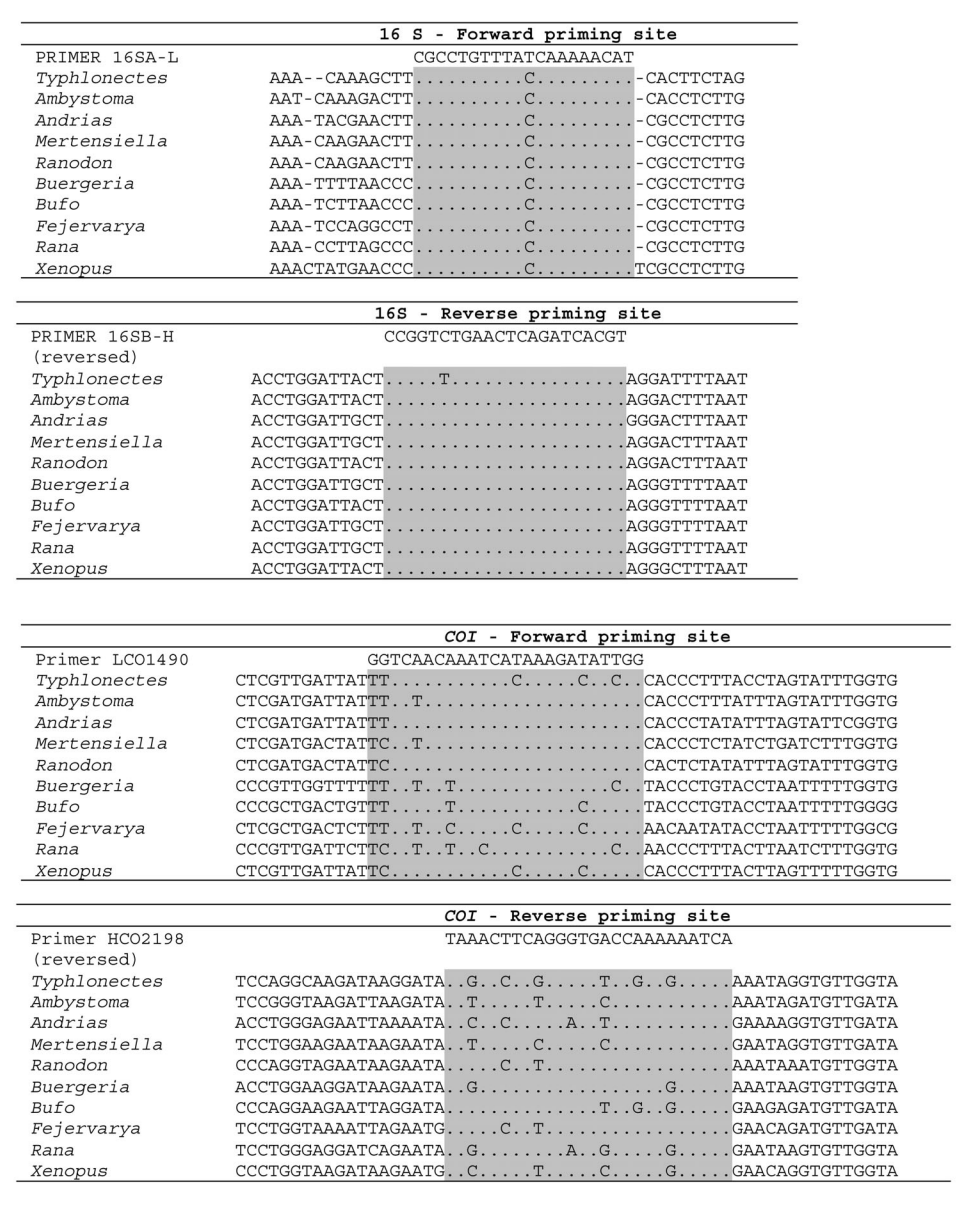
**Variability of priming sites in amphibians. **Variability of priming sites for *16S rRNA *and *COI *in amphibians.

**Figure 2:**
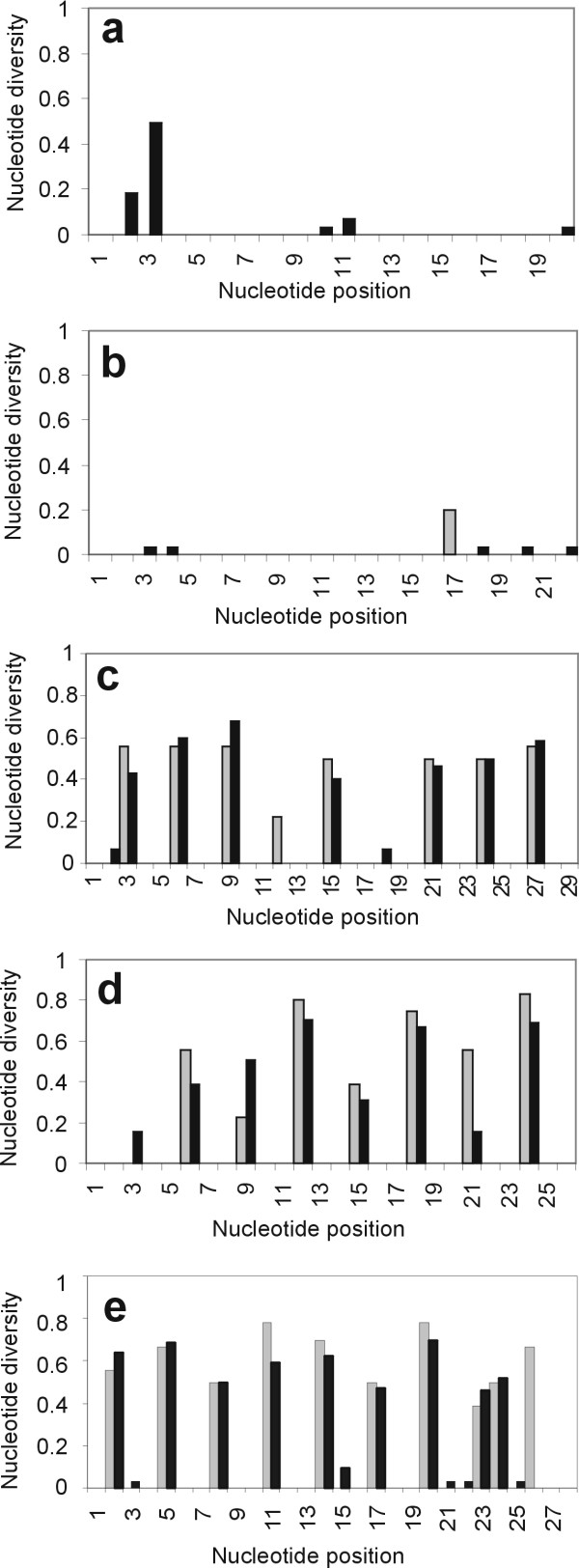
**Variation of priming sites vertebrates. **Variation in priming sites of *16S rRNA *(a, F-primer; b, R-primer) and *COI *(c, Bird-F1, LCO1490; d, HCO2198; e, Bird-R1, Bird-R2) fragments studied herein. Values are nucleotide variability as calculated using the DNAsp program. Grey bars show the values for nine amphibians, black bars the values for a set of 59 other vertebrates (see Materials and Methods, and Figs. 3-4).

The variability of priming sites was surveyed using nine complete amphibian mitochondrial sequences from Genbank (Fig. 1), and 59 mt genomes of fishes, reptiles, birds and mammals (Fig. 2). A high variability was encountered for *COI*. The sequences of some species were largely consistent with the primers: *Xenopus *had two mutations only at each of the priming regions. However, other sequences were strongly different, with up to seven mutations, all at third codon positions. No particular pattern was recognizable for any major group that would facilitate designing *COI *primers specific for frogs, salamanders or caecilians. Interestingly the variability among the amphibian sequences available was as large as or larger than among the complete set of vertebrates at many nucleotide positions of *COI *priming sites (Fig. 2), indicating a possible higher than average variability of this gene in amphibians.

In contrast, the *16S *priming sites were remarkably constant both among amphibians and among other vertebrates (Fig. 1, 2). A wider survey of priming sites, i.e., the alternative reverse priming sites used in arthropod and bird studies [2,7], confirmed the high variability of *COI *in amphibians, and in vertebrates in general (Fig. 2). A screening of the first 800 bp of the C-terminal part of the gene in nine amphibians of which complete mitochondrial genes were available did not reveal a single fragment of 20 bp where all nine species would agree in 80% or more of their nucleotides.

### Recovery of major groups

**Figure 3:**
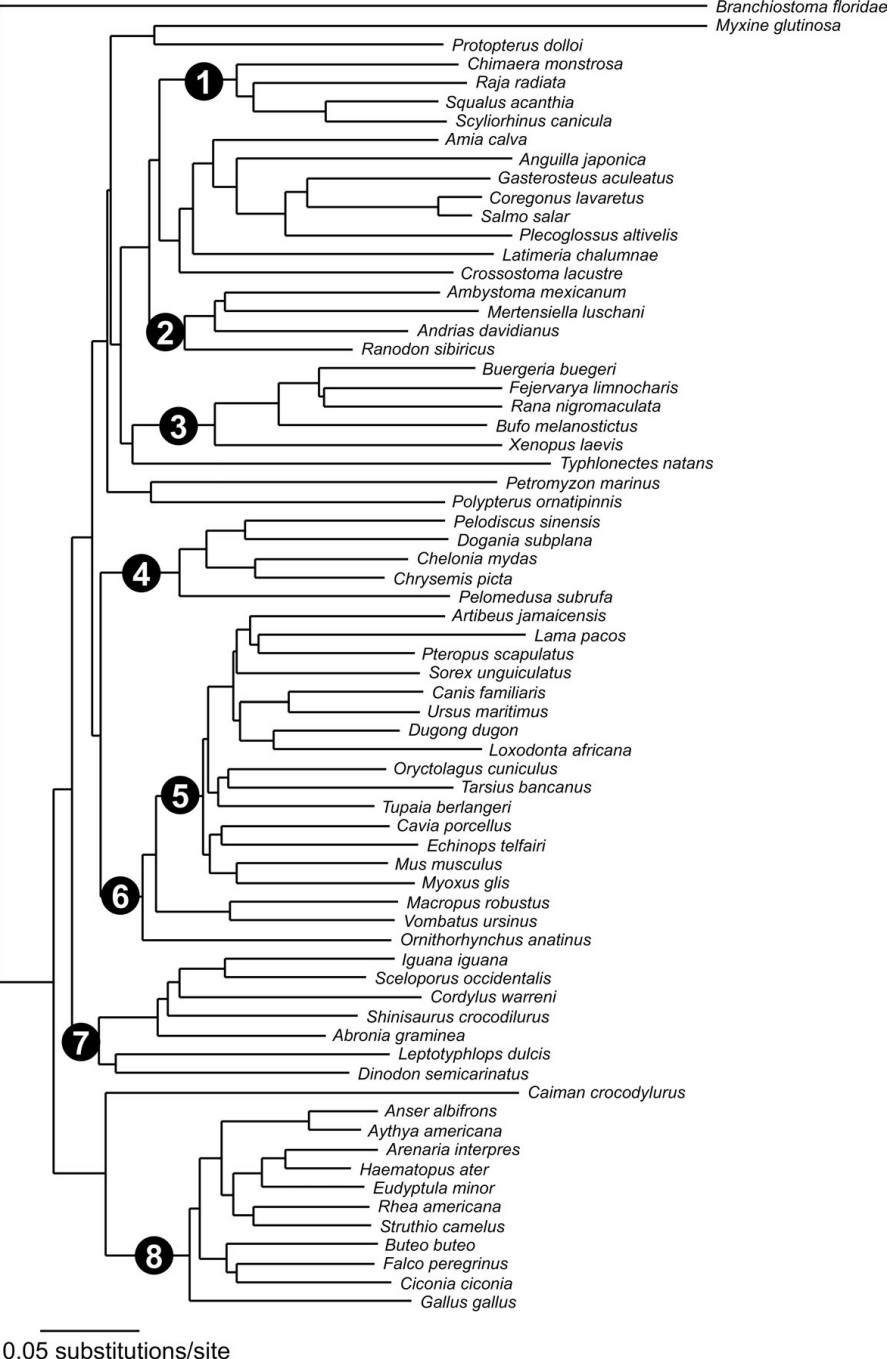
**16S Neighbor-joining tree of selected vertebrate taxa. **Neighbor-joining tree of selected vertebrate taxa based on the fragment of the 16SrRNA gene amplified by primers 16SaL and 16SbH. Numbers in black circles indicate major clades that were recovered by this analysis: (1) cartilaginous fishes, (2) salamanders, (3) frogs, (4) turtles, (5) eutherian mammals, (6) mammals, (7) squamates, (8) birds.

**Figure 4:**
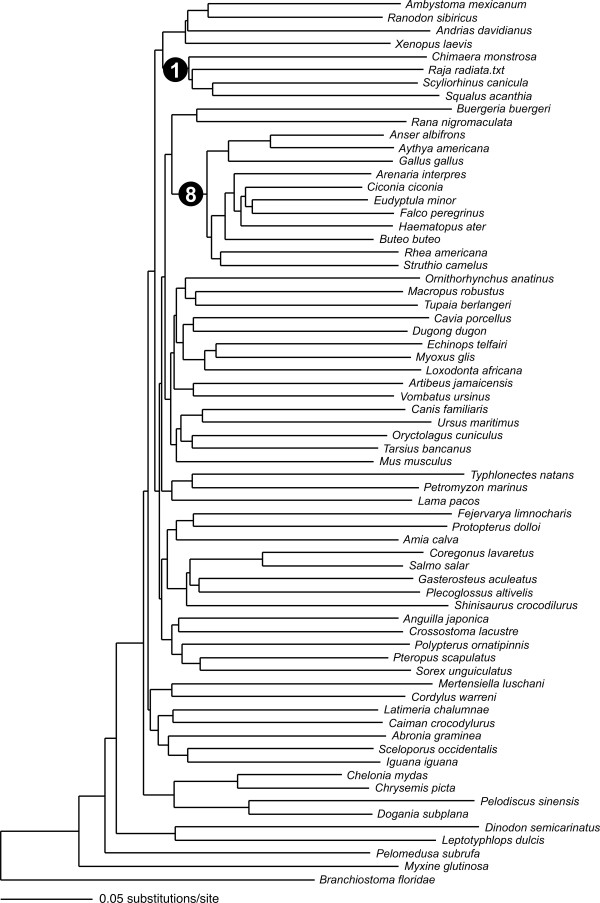
**COI Neighbor-joining tree of selected vertebrate taxa. **Neighbor-joining tree of selected vertebrate taxa based on the fragment of the *COI *gene amplified by primers LCO1490 and HCO2198. Numbers in black circles indicate major clades that were recovered by this analysis, corresponding to the numbering in Supp. material D. Only two of the clades recovered by the *16S *analysis are also monophyletic here: (1) cartilaginous fishes, (8) birds.

The phenetic neighbor-joining analysis using the *16S *fragment produced a tree that contained eight major groupings that conform to or are congruent with the current classification and phylogeny (Fig. 3): cartilaginous fishes, salamanders, frogs, turtles, eutherian mammals, mammals, squamates, birds. Of these, the *COI *tree (Fig. 4) recovered only the lineages of cartilaginuous fishes and birds. The *COI *analysis did not recover any additional major lineage.

### 16S rDNA barcoding of tadpoles

From an ongoing project involving the large-scale identification of tadpoles of Madagascan frogs [5] we here provide data from larval and adult frog species from two sites of high anuran diversity in eastern Madagascar, Andasibe and Ranomafana. These two localities are separated by a geographical distance of ca. 250 km. The results will be presented in more detail elsewhere.

**Figure 5:**
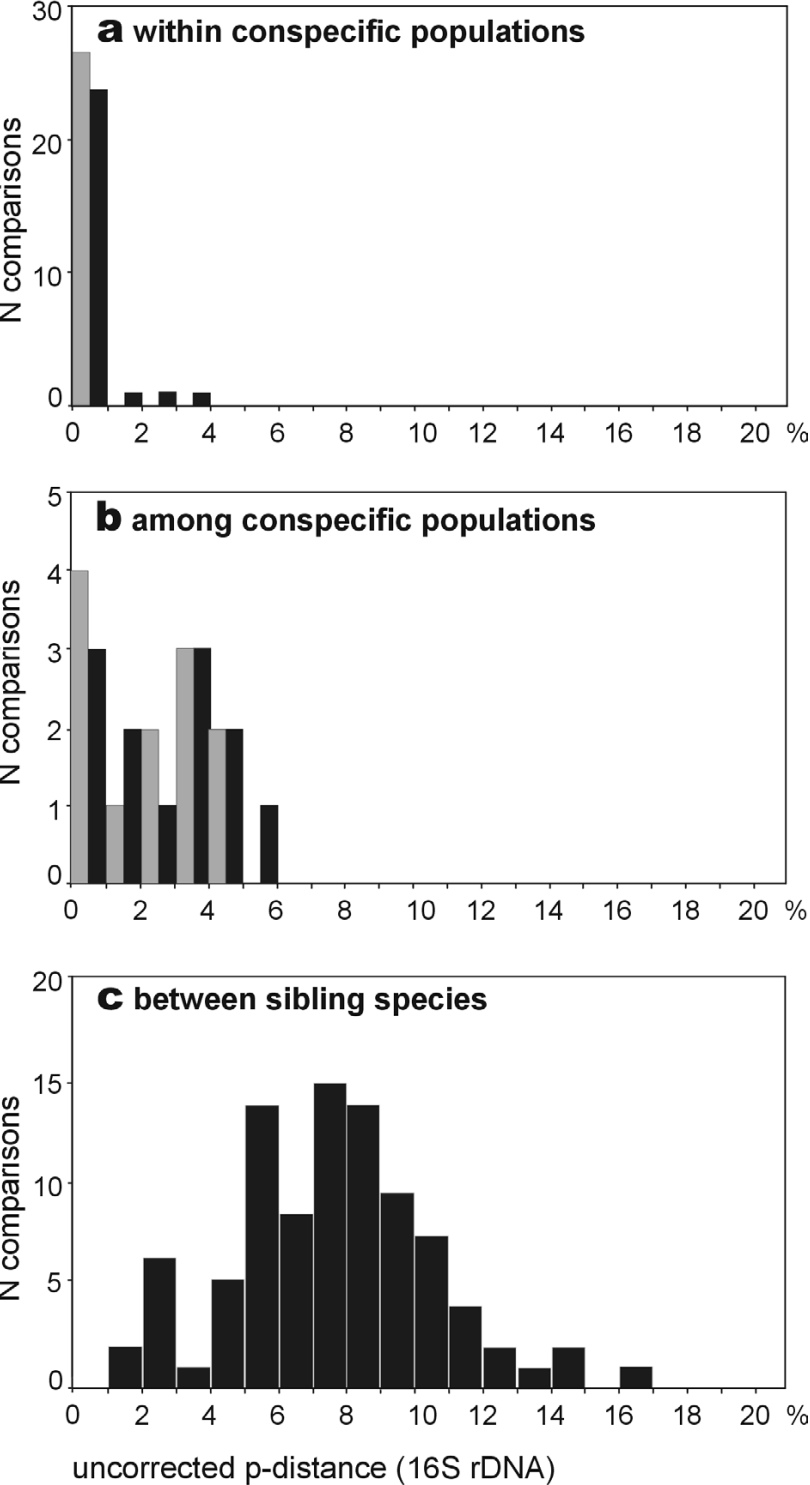
**16S inter- and intraspecific genetic variation in Malagasy frogs. **Variation in the fragment of the 16S rRNA gene (ca. 550 bp) studied herein, (a) within populations, (b) among conspecific populations and (c) among sibling species of frogs in the family Mantellidae from Madagascar. The values are uncorrected p-distances from pairwise comparisons in the respective category. Only one (mean) value per species was used in (a) and (b), even when multiple individuals were compared. Grey bars in (a) and (b) show the mean values from all possible individual comparisons within a species, black bars are the maximum divergences encountered between two individual sequences.

We selected target species for which morphological and bioacoustic uniformity suggests that populations from Ranomafana and Andasibe are conspecific. All these species belong to the family Mantellidae. We then analysed haplotypes within and between these populations. In addition we assessed divergences among sibling species of mantellid frogs (Tables 2-4, Additional file 1). These were defined as morphologically similar species that are phylogenetically sister to each other, or are in well-defined but phylogenetically poorly resolved clades of 3–5 species. Results revealed a low intrapopulational variation of 0–3% uncorrected pairwise distances in the *16S *gene, a surprisingly large differentiation among conspecific populations of 0–5.1%, and a wide range of differentiation among species, ranging from 1–16.5% with a mode at 7–9% (Fig. 5). The few species separated by low genetic distances were allopatrically distributed. The interspecific divergence was higher in those species pairs in which syntopic occurrence has been recorded or is likely (2.7–16.5% divergence, mean 8.5%) as compared to those that so far only have been found in allopatry (1.0–12.9%, mean 6.9%).

Phylogenetic and phenetic analyses (Bayesian and Neighbor-joining) of these and many additional sequences (to be published elsewhere) mostly grouped sequences of those specimens from Ranomafana and Andasibe that a priori had been considered to be conspecific (exceptions were *Mantidactylus boulengeri*, not considered in the intraspecific comparisons here, and *M. blommersae*). This indicates that cases in which haplotypes of a species are more similar to those of another species than to those of other conspecific individuals or populations, are rare in these frogs. Sharing of identical haplotypes among individuals belonging to different species, in our dataset, was limited to three closely related species pairs of low genetic divergences: *Boophis doulioti *and *B. tephraeomystax*, *B. goudoti *and *B*. cf.*periegetes*, *Mantella aurantiaca *and *M. crocea*. Depending on the taxonomic scheme employed, our complete data set contains 200–300 species of Madagascan frogs. Hence, haplotype sharing was demonstrated in 2–3% of the total number of species only.

To explore the reliability of tadpole identification using the *16S *gene we used local BLAST searches against a database containing about 1000 sequences of adult frogs from a wide sampling all over Madagascar. 138 tadpoles from the Andasibe region and 84 tadpoles from the Ranomafana region were compared with adult sequences in the database. In 77% of the cases the highest scores were those from comparisons to adults from the same site as the tadpoles. In most of the unsuccessful comparisons, adult sequences of the corresponding species were not available from the tadpole site (21%). In only 5 cases (2%) conspecific adults collected from a different site than the tadpoles yielded higher BLAST scores although adult sequences from the same site were in the database.

## Conclusion

### DNA barcoding in amphibians

DNA barcoding has great appeal as a universally applicable tool for identification of species and variants of organisms, possibly even in automated handheld devices [14]. However, doubtless severe restrictions exist to its universal applicability [9]. Some taxa, e.g. cichlid fishes of Lake Victoria, have radiated so rapidly that the speciation events have not left any traces in their mitochondrial genomes [15]; identifying these species genetically will only be possible through the examination of multiple nuclear markers, as it has been done to assess their phylogeny [16]. Some snails are characterized by a high intraspecific haplotype diversity, which could disable attempts to identify and distinguish among species using such markers [17].

Haplotype sharing due to incomplete lineage sorting or introgression is also known in amphibians [18] although it was not common in mantellid frogs in our data set. However, a number of species showed haplotype sharing with other species, or non-monophyletic haplotypes, warranting a more extensive discussion. In *Mantidactylus boulengeri*, specimens from Andasibe and Ranomafana have similar advertisement calls and (at least superficially) similar morphologies, but their *16S *haplotypes were not a monophyletic group (unpublished data). This species belongs to a group of direct-developing frogs that, like the Neotropical *Eleutherodactylus *[19] may be characterized by a high rate of cryptic speciation. Further data are necessary to decide whether the populations from Ranomafana and Andasibe are indeed conspecific. In contrast, there is little doubt that the populations of *Mantidactylus blommersae *from these two sites are conspecific, yet the Ranomafana haplotypes are closer to those of the clearly distinct species *M. domerguei*. The species pairs where haplotype sharing has been observed (see Results) all appear to be allopatrically to parapatrically distributed and show no or only low differences in advertisement calls, indicating that occasional hybridization along contact zones may be possible [e.g., [20]]. Haplotypes of each of these species pairs always formed highly supported clusters or clades, and had divergences below 3%, indicating that haplotype sharing in mantellids may only constitute a problem when individuals are to be assigned to such closely related sister species.

Although our data show that DNA barcoding in mantellids is a largely valid approach when both reference and test sequences come from the same site, the occurrence of non-monophyletic and highly divergent haplotypes within species characterizes these and other amphibians as a challenging group for this technique. Certainly, DNA barcoding is unable to provide a fully reliable species identification in amphibians, especially if reference sequences do not cover the entire genetic variability and geographic distribution of a species. However, the same is true for any other morphological or bioacoustic identification method. Case studies are needed to estimate more precisely the margin of error of molecular identification of amphibian species. For many approaches, such as the molecular survey of the trade in frog legs for human consumption [21], the error margins might be acceptable. In contrast, the broad overlap of intraspecific and interspecific divergences (Fig. 5) cautions against simplistic diagnoses of presumably new amphibian species by DNA divergences alone. A large proportion of biological and evolutionary species will be missed by inventories that characterize candidate species by DNA divergences above a previously defined threshold.

### Comparative performance of DNA barcoding markers in amphibians

Phenomena of haplotype sharing or non-monophyletic conspecific haplotypes will affect any DNA barcoding approach that uses mitochondrial genes, and are also to be expected with nuclear genes [e.g., [22]]. Nevertheless, some genes certainly outperform others in terms of discriminatory power and universal applicability, and these characteristics may also vary among organism groups. The mitochondria of plants are characterized by very different evolutionary patterns than those of animals, including frequent translocation of genetic material into and from the nucleus [23], which limits their use for DNA barcoding purposes. Nuclear ribosomal DNA (*18S *and *28S*), proposed as standard marker [3], has a high potential in invertebrate DNA barcoding but its high-throughput amplification encounters difficulties in vertebrates.

As a consequence, despite the need of consensus on markers for universal applicability of DNA barcoding, the use of different genes in different groups of organisms seems reasonable. It has been hypothesized that universal *COI *primers may enable amplification of a 5' terminal fragment from representatives of most animal phyla due to their robustness [2]. The success in DNA barcoding of lepidopterans and birds suggests that this gene fragment can indeed be used as a standard for many higher animal taxa [2,4,7].

In our experiments we compared *16S *primers commonly used in amphibians to *COI *primers that had been developed for other vertebrates [7] or invertebrates [2]. It may well be possible, with some effort, to design primers that are more successful and consistent in amplifying *COI *from amphibians. However, our results from mantellid frogs (Table 1, Additonal file 1) indicate a very good amplification success of the primers for some species, but failure for other, related species. This and our results on variability of priming sites predict enormous difficulties in designing one pair of primers that will reliably amplify this gene fragment in all vertebrates, all amphibians, or even all representatives of any amphibian order. A set of one forward and three reverse *COI *primers have been successfully used to amplify and sequence a large number of bird species [7], but birds are a much younger clade than amphibians with a probably lower mitochondrial variability.

A further optimization of *COI *amplification may also be achieved regarding the PCR protocol. Herein we used standard protocols that optimized annealing temperature only, whereas more complex touchdown protocols have been used for birds and butterflies [4,7]. However, one major requirement for a DNA barcoding marker is its robustness to variable lab conditions. If DNA barcoding is to be applied as a standard in many different labs, primers and genes need to be chosen that amplify reliably under very different conditions and under standard protocols. This clearly applies to *16S*, which we have amplified with very different annealing temperatures and PCR conditions in previous exploratory studies (results not shown).

Alignment of *16S *sequences is complicated by the prevalence of insertions and deletions, and this gene is less variable than *COI *[2]. Nevertheless, our results indicate that even using an uncritical automated alignment this gene has a higher potential than *COI *to assign vertebrate sequences to the level of classes and orders.

The *16S *gene is a highly conserved mitochondrial marker but mutations are common in some variable regions, corresponding to loops in the ribosomal RNA structure. In amphibians, where many species are relatively old entities [24], this ensures a sufficient amount of mutations among species. Our results for amphibians, and previous experience with fishes, reptiles and mammals, indicates that *16S *is sufficiently variable to unambiguously identify most species.

A further mitochondrial gene that has been widely used in amphibian phylogenetic and phylogeographic studies is *cytochrome b*. This gene can easily be amplified in salamanders and archaeobatrachian frogs using primers that anneal with adjacent tRNA genes. However, neobatrachian frogs (the wide majority of amphibian species) are characterized by rearrangements of the mitochondrial genome [25,26], and *cytochrome b *in these species borders directly to the control region. Although *cytochrome b *primers are available that work in many neobatrachians [27,28], they are not fully reliable. According to our own observations in mantellid frogs, these primers may amplify this gene in one species but fail in other closely related species, presumably because of mutations at the priming sites and similar to the *COI *primers tested here.

In contrast, the *16S *primer pair used here can be considered as truly universal not only for amphibians but even for vertebrates. This is also reflected by the high number of amphibian *16S *sequences in Genbank (2620 hits for *16S *vs. 483 hits for *COI*, as of September 2004). Moreover, the *16S *and *12S *rRNA genes have been selected as standard markers for phylogeny reconstruction in amphibians [29], which will lead to a near-complete global dataset of amphibian 16S sequences in the near future. If the development of handheld devices [14] is envisaged as a realistic goal, then the universality and robustness of primers should be among the most relevant characteristics of a gene for DNA barcoding. When pooled samples containing representatives of various higher vertebrate taxa are to be analysed, the risk of false negatives strongly increases with decreasing universality of primers. As a consequence we recommend the use of *16S *as additional standard DNA barcoding marker for vertebrates, especially for but not limited to applications that involve pooled samples.

## Methods

To test for universality of primers and cycling conditions, we performed parallel experiments in three different laboratories (Berkeley, Cologne, Konstanz) using the same primers but different biochemical products and thermocyclers, and slightly different protocols.

The selected primers for *16S *[30] amplify a fragment of ca. 550 bp (in amphibians) that has been used in many phylogenetic and phylogeographic studies in this and other vertebrate classes: 16SA-L, 5' - CGC CTG TTT ATC AAA AAC AT - 3'; 16SB-H, 5' - CCG GTC TGA ACT CAG ATC ACG T - 3'.

For *COI *we tested (1) three primers designed for birds [7] that amplify a 749 bp region near the 5'-terminus of this gene: BirdF1, 5' - TTC TCC AAC CAC AAA GAC ATT GGC AC - 3', BirdR1, 5' - ACG TGG GAG ATA ATT CCA AAT CCT G - 3', and BirdR2, 5' - ACT ACA TGT GAG ATG ATT CCG AAT CCA G - 3'; and (2) one pair of primers designed for arthropods [2] that amplify a 658 bp fragment in the same region: LCO1490, 5' - GGT CAA CAA ATC ATA AAG ATA TTG G - 3', and HCO2198, 5'-TAA ACT TCA GGG TGA CCA AAA AAT CA-3'. Sequences of additional primers for *COI *that had performed well in mammals and fishes were kindly made available by P. D. N. Hebert (personal communication in 2004) and these primers yielded similar results (not shown).

The optimal annealing temperatures for the *COI *primers were determined using a gradient thermocycler and were found to be 49–50°C; the 16S annealing temperature was 55°C. Successfully amplified fragments were sequenced using various automated sequencers and deposited in Genbank. Accession numbers for the complete data set of adult mantellid sequences used for the assessment of intra- and interspecific divergences (e.g. in Fig. 5) are AY847959–AY848683. Accession numbers of the obtained COI sequences are AY883978–AY883995.

Nucleotide variability was scored using the software DNAsp [31] at *COI *and *16S *priming sites of the following complete mitochondrial genomes of nine amphibians and 59 other vertebrates: **Cephalochordata: **AF098298, *Branchiostoma*. **Myxiniformes: **AJ404477, *Myxine*. Petromyzontiformes: U11880, *Petromyzon*. **Chondrichthyes**: AJ310140, *Chimaera*; AF106038, *Raja*; Y16067, *Scyliorhinus*; Y18134, *Squalus*. **Actinopterygii**: AY442347, *Amia*; AB038556, *Anguilla*; AB034824, *Coregonus*; M91245, *Crossostoma*; AP002944, *Gasterosteus*; AB047553, *Plecoglossus*; U62532, *Polypterus*; U12143, *Salmo*. **Dipnoi**: L42813, *Protopterus*. **Coelacanthiformes**: U82228, *Latimeria*. **Amphibia, Gymnophiona**: AF154051, *Typhlonectes*. **Amphibia, Urodela**: AJ584639, *Ambystoma*; AJ492192, *Andrias*; AF154053, *Mertensiella*; AJ419960, *Ranodon*. **Amphibia, Anura**: AB127977, *Buergeria*; NC_005794, *Bufo*; AY158705; *Fejervarya*; AB043889, *Rana*; M10217, *Xenopus*. **Testudines**: AF069423, NC_000886, *Chelonia*; *Chrysemys*; AF366350, *Dogania*; AY687385, *Pelodiscus*; AF039066, *Pelomedusa*. **Squamata**: NC_005958, *Abronia*; AB079613, *Cordylus*; AB008539, *Dinodon*; AJ278511, *Iguana*; AB079597, *Leptotyphlops*; AB079242, *Sceloporus*; AB080274, *Shinisaurus*. **Crocodilia**: AJ404872, *Caiman*. **Aves**: AF363031, *Anser*; AY074885, *Arenaria*; AF090337, *Aythya*; AF380305, *Buteo*; AB026818, *Ciconia*; AF362763, *Eudyptula*; AF090338, *Falco*; AY235571, *Gallus*; AY074886, *Haematopus*; AF090339, *Rhea*; Y12025, *Struthio*. **Mammalia**: X83427, *Ornithorhynchus*; Y10524, *Macropus*; AJ304826, *Vombatus*; AF061340, *Artibeus*; U96639, *Canis*; AJ222767, *Cavia *; AY075116, *Dugong*; AB099484, *Echinops*; Y19184, *Lama*; AJ224821, *Loxodonta*; AB042432, *Mus*; AJ001562, *Myoxus*; AJ001588, *Oryctolagus*; AF321050, *Pteropus*; AB061527, *Sorex*; AF348159, *Tarsius*; AF217811, *Tupaia*; AF303111, *Ursus *(for species names, see Genbank under the respective accession numbers).

*16S *sequences of a large sample of Madagascan frogs were used to build a database in Bioedit [32]. Tadpole sequences were compared with this database using local BLAST searches [33] as implemented in Bioedit.

The performance of *COI *and *16S *in assigning taxa to inclusive major clades was tested based on gene fragments homologous to those amplified by the primers used herein (see above), extracted from the complete mitochondrial sequences of 68 vertebrate taxa. Sequences were aligned in Sequence Navigator (Applied Biosystems) by a Clustal algorithm with a gap penalty of 50, a gap extend penalty of 10 and a setting of the ktup parameter at 2. PAUP* [34] was used with the neighbor-joining algorithm and LogDet distances and excluding pairwise comparisons for gapped sites. We chose these simple phenetic methods instead of maximum likelihood or maximum parsimony approaches because they are computationally more demanding and because the aim of DNA barcoding is a robust and fast identification of taxa rather than an accurate determination of their phylogenetic relationships.

## Authors' contributions

MV designed the study and drafted the manuscript. MT performed parts of the PCR experiments and carried out the molecular identifications of tadpoles. AVDM and DRV performed part of the PCR experiments. YC provided results on 16S differentiation among Madagascan frogs. All authors read and approved the final manuscript.

## Supplementary Material

Additional File 1 Summary of results of amplification experiments, and detailed data of inter- and intraspecific divergences in mantellid frogs. Click here for file
