# Supplementary material for: Comparative performance of the 16S rRNA gene in DNA barcoding of amphibians
Source: Front Zool. 2005 Mar 16;2:5. doi: 10.1186/1742-9994-2-5 (PMC555853; doi:10.1186/1742-9994-2-5)
Supplement: Additional File 1 — Summary of results of amplification experiments, and detailed data of inter- and intraspecific divergences in mantellid frogs. [file 1742-9994-2-5-S1.doc]

**Comparative performance of the 16S rRNA gene in DNA barcoding of amphibians**

Miguel Vences^1*^, Meike Thomas^2^, Arie van der Meijden^3^, Ylenia Chiari^3^, David R. Vieites^4^

**Additional Material**

**Additional Material, Table 1.** Taxa of the frog family Mantellidae successfully amplified for *COI* and *16S*. + successful amplification (strong bands on agarose gel), (+) moderate success (faint bands), - no success of amplification. Three primer combinations were simultaneously tested for *COI*. Voucher specimens deposited in the Université d'Antananarivo, Département de Biologie Animale, Madagascar (UADBA) and the Zoological Museum Amsterdam (ZMA). Further collection acronyms used: FAZC, Franco Andreone Zoological Collection, FGMV, collection of Frak Glaw and Miguel Vences.

| **Species** | **Family** | **Vouchers** | ***COI* (BirdF1-BirdR1)** | ***COI* (BirdF1-BirdR2)** | ***COI* (LCO1490 - HCO2198)** | ***16S*** |
| --- | --- | --- | --- | --- | --- | --- |
| **Anura** |  |  |  |  |  |  |
| *Aglyptodactylus madagascariensis* | Mantellidae | FGMV 2002.860, 858 | - | - | (+) | + |
| *Boophis blommersae* | Mantellidae | FGMV 2002.925, 2001.E52 | + | - | - | + |
| *B. goudoti* | Mantellidae | ZMA 19545, 19544 | - | - | + | + |
| *B. marojezensis* | Mantellidae | FAZC 11467, 11483 | - | - | + | + |
| *B. microtympanum* | Mantellidae | UADBA 21178, ZMA 19555 | - | + | - | + |
| *B.aff. ankaratra* | Mantellidae | FAZC 11462, 11480 | + | + | - | + |
| *B. tephraeomystax* | Mantellidae | UADBA 20564, 20623 | - | - | - | + |
| *B. septentrionalis* | Mantellidae | FGMV 2002.915, 914 | - | - | - | + |
| *Mantella betsileo* | Mantellidae | FGMV 2000F34, 2000D62 | + | + | + | + |
| *Mantidactylus asper* | Mantellidae | UADBA 20665, ZMA 19484 | + | + | + | + |
| *M. brevipalmatus* | Mantellidae | UADBA 21184, 21181 | + | + | + | + |
| *M.enki* | Mantellidae | UADBA 20680, ZMA 19436 | - | - | + | + |
| *M. cf. femoralis* | Mantellidae | UADBA 21096, ZMA 19470 | (+) | + | + | + |
| *M. grandidieri* | Mantellidae | UADBA 20612, 20584 | - | - | + | + |
| *M. melanopleura* | Mantellidae | no voucher | - | - | + | + |
| *M. tschenki* | Mantellidae | UADBA 20641, ZMA 19392 | + | (+) | - | + |
| *M. ulcerosus* | Mantellidae | FGMV 2002.449, 2002.546 | + | + | + | + |
| *M. sculpturatus* | Mantellidae | UADBA 20605, 20617 | + | + | + | + |
| *M. aff. wittei* | Mantellidae | FGMV 2002.880. 2002.879 | - | - | + | + |
| *Ptychadena mascariensis* | Ranidae | no voucher | (+) | + | + | + |
| **Other vertebrates** |  |  |  |  |  | + |
| *Furcifer pardalis* | [Squamata] | no voucher | + | + | + | + |
| *Mus musculus* | [Mammalia] | no voucher | - | - | (+) | + |

**Additional Material, Table 2.** Uncorrected pairwise p-distances (=observed percent of sequence

difference) within populations of mantellid frogs from Madagascar. N is the number of sequenced individuals. The table gives mean values over all pairwise comparisons among the individuals of one population, and minimum and maximum values recorded. For the purpose of better graphical representation in Fig. 5, the original p-distances as shown below were transformed into percentages (*100). Asterisks mark populations which are from the type locality, or from very nearby, and can therefore reliably be considered as reference sequences for the corresponding species.

| **Species** | **N** | **Locality** | **Mean** | **Min** | **Max** |
| --- | --- | --- | --- | --- | --- |
| *Aglyptodactylus madagascariensis* | 6 | Andasibe | 0.0019 | 0.0000 | 0.0043 |
| *Boophis rappiodes* | 7 | Andasibe | 0.0026 | 0.0000 | 0.0039 |
| **Boophis viridis* | 9 | Andasibe | 0.0014 | 0.0000 | 0.0042 |
| **Mantidactylus blommersae* | 8 | Andasibe | 0.0015 | 0.0000 | 0.0040 |
| **Mantidactylus liber* | 5 | Andasibe | 0.0050 | 0.0019 | 0.0077 |
| *Mantidactylus asper* | 8 | Andasibe (Mandraka) | 0.0003 | 0.0000 | 0.0023 |
| *Mantidactylus redimitus* | 4 | Andasibe (Vohidrazana) | 0.0013 | 0.0000 | 0.0026 |
| *Mantidactylus sculpturatus* | 2 | Andasibe | 0.0000 | 0.0000 | 0.0000 |
| *Aglyptodactylus madagascariensis* | 4 | Ranomafana | 0.0019 | 0.0000 | 0.0038 |
| *Boophis rappiodes* | 5 | Ranomafana | 0.0024 | 0.0000 | 0.0059 |
| *Mantidactylus blommersae* | 5 | Ranomafana | 0.0062 | 0.0000 | 0.0146 |
| *Mantidactylus liber* | 6 | Ranomafana | 0.0023 | 0.0000 | 0.0039 |
| **Mantidactylus enki* | 6 | Ranomafana | 0.0003 | 0.0000 | 0.0023 |
| *Mantidactylus asper* | 4 | Ranomafana | 0.0000 | 0.0000 | 0.0000 |
| *Mantidactylus redimitus* | 6 | Ranomafana | 0.0069 | 0.0000 | 0.0300 |
| *Mantidactylus sculpturatus* | 9 | Ranomafana | 0.0013 | 0.0000 | 0.0023 |
| *Boophis blommersae* | 5 | Tsaratanana | 0.0000 | 0.0000 | 0.0000 |
| *Boophis tephraeomystax* | 6 | Nosy Be | 0.0013 | 0.0000 | 0.0041 |
| *Boophis tephraeomystax* | 5 | Ranomafana | 0.0000 | 0.0000 | 0.0000 |
| *Boophis doulioti* | 2 | Kirindy | 0.0000 | 0.0000 | 0.0000 |
| **Mantidactylus wittei* | 4 | Nosy Be | 0.0010 | 0.0000 | 0.0021 |
| **Mantella aurantiaca* | 10 | Moramanga etc. | 0.0095 | 0.0000 | 0.0230 |
| **Mantidactylus silvanus* | 6 | Andranofotsy | 0.0010 | 0.0000 | 0.0025 |
| **Mantidactylus pseudoasper* | 3 | Manongarivo | 0.0015 | 0.0000 | 0.0023 |
| *Mantidactylus cf. curtus* | 6 | Andringitra | 0.0011 | 0.0000 | 0.0019 |
| *Mantidactylus charlotteae* | 5 | Andranofotsy | 0.0017 | 0.0000 | 0.0045 |
| *Mantidactylus brevipalmatus* | 3 | Antoetra | 0.0000 | 0.0000 | 0.0000 |

**Additional Material, Table 3.** Uncorrected pairwise p-distances between populations of mantellid frogs from Madagascar. The data are based on comparisons of all individuals from the Andasibe region to all individuals from the Ranomafana region (see supplementary table 2). N is the number of sequenced individuals from both populations. The table gives mean values over all pairwise comparisons, and minimum and maximum values recorded. For the purpose of better graphical representation in Fig. 5, the original p-distances as shown below were transformed into percentages (*100).

| **Species** | **N** | **Mean** | **Min** | **Max** |
| --- | --- | --- | --- | --- |
| *Aglyptodactylus madagascariensis* | 10 | 0.0085 | 0.0062 | 0.0120 |
| *Boophis rappiodes* | 12 | 0.0354 | 0.0334 | 0.0373 |
| *Mantidactylus blommersae* | 13 | 0.0395 | 0.0381 | 0.0422 |
| *Mantidactylus liber* | 11 | 0.0445 | 0.0388 | 0.0511 |
| *Mantidactylus asper* | 12 | 0.0475 | 0.0458 | 0.0481 |
| *Mantidactylus redimitus* | 10 | 0.0253 | 0.0000 | 0.0332 |
| *Mantidactylus sculpturatus* | 11 | 0.0259 | 0.0247 | 0.0271 |
| *Boophis guibei* | 2 | 0.0150 | 0.0150 | 0.0150 |
| *Mantidactylus pulcher* | 2 | 0.0057 | 0.0057 | 0.0057 |
| *Mantidactylus depressiceps* | 2 | 0.0314 | 0.0314 | 0.0314 |
| *Mantidactylus malagasius* | 2 | 0.0090 | 0.0090 | 0.0090 |
| *Mantidactylus argenteus* | 2 | 0.0000 | 0.0000 | 0.0000 |

**Additional Material, Table 4.** Uncorrected pairwise p-distances between sibling species of mantellid frogs. The table gives mean values over all pairwise comparisons of individuals sequenced from the two species compared (maximum and minimum values showed no relevance differences to these values). Sibling species were defined as morphologically similar species that are phylogenetically sister to each other, or are in well-defined but phylogenetically poorly resolved clades of 3-5 species. The last column indicates whether syntopic occurence of the two compared species has been recorded. A Yes in parentheses indicates cases where syntopy has not been fully ascertained but is likely due to either unconfirmed call records, or broadly sympatric occurrence with similar habitat requirements. For the purpose of better graphical representation in Fig. 5, the original p-distances as shown below were transformed into percentages (*100).

| **Species 1** | **Species 2** | **Mean p-distance** | **Syntopy recorded?** |
| --- | --- | --- | --- |
| *Boophis andreonei* | *Boophis aff elenae* | 0.0856 | - |
| *Boophis doulioti* | *Boophis tephraeomystax* | 0.0242 | - |
| *Boophis erythrodactylus* | *Boophis bottae* | 0.0870 | - |
| *Boophis idae* | *Boophis guibei* | 0.0589 | Yes |
| *Boophis jaegeri* | *Boophis aff elenae* | 0.0990 | - |
| *Boophis jaegeri* | *Boophis andreonei* | 0.0933 | - |
| *Boophis luteus* | *Boophis aff elenae* | 0.1006 | Yes |
| *Boophis luteus* | *Boophis andreonei* | 0.0945 | (Yes) |
| *Boophis luteus* | *Boophis jaegeri* | 0.1087 | - |
| *Boophis majori* | *Boophis feonnyala* | 0.1156 | - |
| *Boophis mandraka* | *Boophis liami* | 0.0795 | - |
| *Boophis marojezensis* | *Boophis feonnyala* | 0.1404 | (Yes) |
| *Boophis marojezensis* | *Boophis majori* | 0.1648 | Yes |
| *Boophis picturatus* | *Boophis feonnyala* | 0.0885 | (Yes) |
| *Boophis picturatus* | *Boophis majori* | 0.1136 | Yes |
| *Boophis picturatus* | *Boophis marojezensis* | 0.1342 | Yes |
| *Boophis pyrrhus* | *Boophis feonnyala* | 0.1228 | Yes |
| *Boophis pyrrhus* | *Boophis majori* | 0.1291 | - |
| *Boophis pyrrhus* | *Boophis marojezensis* | 0.1488 | (Yes) |
| *Boophis pyrrhus* | *Boophis picturatus* | 0.1182 | (Yes) |
| *Boophis rappiodes* | *Boophis bottae* | 0.1021 | Yes |
| *Boophis rappiodes* | *Boophis erythrodactylus* | 0.1078 | - |
| *Boophis schuboeae* | *Boophis ankaratra* | 0.0284 | - |
| *Boophis septentrionalis* | *Boophis aff elenae* | 0.0759 | - |
| *Boophis septentrionalis* | *Boophis andreonei* | 0.0701 | - |
| *Boophis septentrionalis* | *Boophis jaegeri* | 0.0875 | - |
| *Boophis septentrionalis* | *Boophis luteus* | 0.0825 | - |
| *Boophis sibilans* | *Boophis aff sibilans* | 0.0758 | (Yes) |
| *Boophis solomaso* | *Boophis liami* | 0.0849 | (Yes) |
| *Boophis solomaso* | *Boophis mandraka* | 0.0795 | - |
| *Boophis tasymena* | *Boophis bottae* | 0.1179 | (Yes) |
| *Boophis tasymena* | *Boophis erythrodactylus* | 0.0968 | - |
| *Boophis tasymena* | *Boophis rappiodes* | 0.0856 | (Yes) |
| *Mantella aurantiaca* | *Mantella crocea* | 0.0115 | - |
| *Mantidactylus ambreensis* | *Mantidactylus femoralis* | 0.0768 | - |
| *Mantidactylus ambreensis* | *Mantidactylus mocquardi* | 0.0827 | - |
| *Mantidactylus ambreensis* | *Mantidactylus zolitschka* | 0.0806 | - |
| *Mantidactylus boulengeri* | *Mantidactylus blanci* | 0.0631 | - |
| *Mantidactylus brevipalmatus* | *Mantidactylus delormei* | 0.0402 | - |
| *Mantidactylus brevipalmatus* | *Mantidactylus aerumnalis* | 0.0840 | - |
| *Mantidactylus cf .pauliani (Itremo)* | *Mantidactylus pauliani* | 0.0702 | - |
| *Mantidactylus cf thelenae* | *Mantidactylus blanci* | 0.0726 | - |
| *Mantidactylus cf thelenae* | *Mantidactylus boulengeri* | 0.0703 | Yes |
| *Mantidactylus cf. biporus*  *(Manongarivo 2)* | *Mantidactylus cf. biporus*  *(Manongarivo 1)* | 0.0345 | - |
| *Mantidactylus cf. curtus (Itremo)* | *Mantidactylus madecassus* | 0.0989 | (Yes) |
| *Mantidactylus cf. curtus (Itremo)* | *Mantidactylus pauliani* | 0.1048 | - |
| *Mantidactylus cf. curtus (Itremo)* | *Mantidactylus cf. pauliani (Itremo)* | 0.0873 | Yes |
| *Mantidactylus cf. pauliani (Itremo)* | *Mantidactylus madecassus* | 0.0492 | - |
| *Mantidactylus charlottae* | *Mantidactylus albofrenatus* | 0.1020 | - |
| *Mantidactylus corvus* | *Mantidactylus pseudoasper* | 0.0767 | - |
| *Mantidactylus curtus* | *Mantidactylus cf. pauliani (Itremo)* | 0.0102 | - |
| *Mantidactylus decaryi* | *Mantidactylus blanci* | 0.0541 | Yes |
| *Mantidactylus decaryi* | *Mantidactylus boulengeri* | 0.0586 | (Yes) |
| *Mantidactylus decaryi* | *Mantidactylus cf thelenae* | 0.0590 | - |
| *Mantidactylus domerguei* | *Mantidactylus blommersae* | 0.0482 | Yes |
| *Mantidactylus enki* | *Mantidactylus blanci* | 0.0270 | Yes |
| *Mantidactylus enki* | *Mantidactylus boulengeri* | 0.0495 | (Yes) |
| *Mantidactylus enki* | *Mantidactylus cf thelenae* | 0.0612 | - |
| *Mantidactylus enki* | *Mantidactylus decaryi* | 0.0428 | (Yes) |
| *Mantidactylus grandisonae* | *Mantidactylus blommersae* | 0.0927 | Yes |
| *Mantidactylus grandisonae* | *Mantidactylus domerguei* | 0.0996 | Yes |
| *Mantidactylus granulatus* | *Mantidactylus leucomaculatus* | 0.0734 | Yes |
| *Mantidactylus kathrinae* | *Mantidactylus depressiceps* | 0.0783 | (Yes) |
| *Mantidactylus leucocephalus* | *Mantidactylus blanci* | 0.0676 | (Yes) |
| *Mantidactylus leucocephalus* | *Mantidactylus boulengeri* | 0.0631 | - |
| *Mantidactylus leucocephalus* | *Mantidactylus cf thelenae* | 0.0522 | - |
| *Mantidactylus leucocephalus* | *Mantidactylus decaryi* | 0.0270 | - |
| *Mantidactylus leucocephalus* | *Mantidactylus enki* | 0.0518 | - |
| *Mantidactylus massi* | *Mantidactylus aglavei* | 0.0858 | - |
| *Mantidactylus mocquardi* | *Mantidactylus femoralis* | 0.0746 | Yes |
| *Mantidactylus pauliani* | *Mantidactylus madecassus* | 0.0511 | - |
| *Mantidactylus phantasticus* | *Mantidactylus aglavei* | 0.0655 | Yes |
| *Mantidactylus phantasticus* | *Mantidactylus massi* | 0.0292 | - |
| *Mantidactylus redimitus* | *Mantidactylus cornutus* | 0.0769 | Yes |
| *Mantidactylus sarotra* | *Mantidactylus blommersae* | 0.1046 | Yes |
| *Mantidactylus sarotra* | *Mantidactylus kely* | 0.0688 | - |
| *Mantidactylus striatus* | *Mantidactylus malagasius* | 0.0562 | (Yes) |
| *Mantidactylus tandroka* | *Mantidactylus salegy* | 0.0516 | - |
| *Mantidactylus tornieri* | *Mantidactylus depressiceps* | 0.0902 | Yes |
| *Mantidactylus tornieri* | *Mantidactylus kathrinae* | 0.0559 | (Yes) |
| *Mantidactylus tschenki* | *Mantidactylus cornutus* | 0.0240 | - |
| *Mantidactylus tschenki* | *Mantidactylus redimitus* | 0.0674 | (Yes) |
| *Mantidactylus wittei* | *Mantidactylus blommersae* | 0.0903 | - |
| *Mantidactylus wittei* | *Mantidactylus sp (Mayotte)* | 0.0524 | - |
| *Mantidactylus zavona* | *Mantidactylus leucomaculatus* | 0.0831 | - |
| *Mantidactylus zavona* | *Mantidactylus granulatus* | 0.0671 | Yes |
| *Mantidactylus zolitschka* | *Mantidactylus femoralis* | 0.0553 | Yes |
| *Mantidactylus zolitschka* | *Mantidactylus mocquardi* | 0.0552 | Yes |
